# When Bigger Is Better: 3D RNA Profiling of the Developing Head in the Catshark *Scyliorhinus canicula*

**DOI:** 10.3389/fcell.2021.744982

**Published:** 2021-10-22

**Authors:** Hélène Mayeur, Maxence Lanoizelet, Aurélie Quillien, Arnaud Menuet, Léo Michel, Kyle John Martin, Sébastien Dejean, Patrick Blader, Sylvie Mazan, Ronan Lagadec

**Affiliations:** ^1^CNRS, Sorbonne Université, UMR 7232-Biologie Intégrative des Organismes Marins (BIOM), Observatoire Océanologique, Banyuls sur Mer, France; ^2^Molecular, Cellular and Developmental Biology (MCD UMR 5077), Centre de Biologie Intégrative (CBI, FR 3743), Université de Toulouse, CNRS, UPS, Toulouse, France; ^3^UMR 7355, Experimental and Molecular Immunology and Neurogenetics, CNRS and University of Orléans, Orléans, France; ^4^United Kingdom Research and Innovation, Biotechnology and Biological Sciences Research Council, Swindon, United Kingdom; ^5^Institut de Mathématiques de Toulouse, Université de Toulouse, CNRS, UPS, UMR 5219, Toulouse, France

**Keywords:** RNA tomography, catshark, forebrain patterning, correlation, auto-correlation

## Abstract

We report the adaptation of RNA tomography, a technique allowing spatially resolved, genome-wide expression profiling, to a species occupying a key phylogenetic position in gnathostomes, the catshark *Scyliorhinus canicula*. We focused analysis on head explants at an embryonic stage, shortly following neural tube closure and of interest for a number of developmental processes, including early brain patterning, placode specification or the establishment of epithalamic asymmetry. As described in the zebrafish, we have sequenced RNAs extracted from serial sections along transverse, horizontal and sagittal planes, mapped the data onto a gene reference taking advantage of the high continuity genome recently released in the catshark, and projected read counts onto a digital model of the head obtained by confocal microscopy. This results in the generation of a genome-wide 3D atlas, containing expression data for most protein-coding genes in a digital model of the embryonic head. The digital profiles obtained for candidate forebrain regional markers along antero-posterior, dorso-ventral and left-right axes reproduce those obtained by *in situ* hybridization (ISH), with expected relative organizations. We also use spatial autocorrelation and correlation as measures to analyze these data and show that they provide adequate statistical tools to extract novel expression information from the model. These data and tools allow exhaustive searches of genes exhibiting any predefined expression characteristic, such a restriction to a territory of interest, thus providing a reference for comparative analyses across gnathostomes. This methodology appears best suited to species endowed with large embryo or organ sizes and opens novel perspectives to a wide range of evo-devo model organisms, traditionally counter-selected on size criterion.

## Introduction

Unraveling spatially resolved gene expression information is crucial for addressing a variety of developmental biology problems, including patterning, regional identity specification, and determination. These questions are currently revolutionized by emerging RNA-seq approaches and concomitantly developing analytical methods aimed at extracting relevant information from the resulting datasets (Lee, 2017; Sun et al., 2020; Waylen et al., 2020). One such approach is RNA tomography or tomo-seq, which was initially applied to the developing zebrafish and mouse (Junker et al., 2014). Briefly, this method relies on the sequencing of frozen sections along three orthogonal planes and subsequent reconstruction of a spatially resolved genome-wide RNA profile, using the iterative proportional fitting algorithm. This results in the generation of a digital 3D model of the tissue of interest, containing genome-wide expression information for every voxel of the model.

While the sequencing of sections has been successfully used for the identification of genes exhibiting regionalized expression along a polarity axis, 3D reconstructions using tomo-seq have only been obtained in the zebrafish and mouse thus far to our knowledge (Junker et al., 2014). We have tested the possibility to conduct this approach in a representative chondrichthyan, the catshark *Scyliorhinus canicula*. As a member of the sister group of osteichthyans, this species occupies a key phylogenetic position to address the origin of gnathostomes from an evo-devo perspective. Its analysis has thus provided insights into gnathostome ancestral features and gene regulatory network modifications, occurring concomitantly with the rise of gnathostome innovations, such as paired limbs, true teeth or articulated jaws, and helped to clarify ancestral features, when discrepancies are observed between actinopterygians and sarcopterygians (Coolen et al., 2008). It is also amenable to experimental approaches during development and while genome size (4.2 Gb) has long been an obstacle to whole genome sequencing projects, an annotated, chromosome-level genome *de novo* assembly has been recently released in this species.

We have focused on the embryonic head at stage 17, which shortly follows neural tube closure. A number of developmental processes of interest, such as the partitioning of the forebrain into its broad subdivisions (Santos-Durán et al., 2015), the appearance of the earliest diencephalic asymmetries (Lagadec et al., 2015), the specification of cephalic mesoderm components (Derobert et al., 2002), or the initiation of optic cup and otic placode formation (Plouhinec et al., 2005; O’Neill et al., 2007), indeed take place in the developing head at this stage. Here, we report the generation of a genome-wide 3D profile of the cephalic region at this stage, as well as the use of correlation and other statistical analysis tools to extract novel information from this model. In addition to providing reference expression data at the stage analyzed, this analysis validates the catshark as a relevant organism for tomo-seq characterizations of embryogenesis.

## Materials and Methods

### Explant Dissection, Sectioning and RNA Extraction From Sections

Eggs from the catshark *S. canicula* were obtained from the Aquariology Service of the Banyuls sur Mer Oceanological Observatory. Stage 17 embryos (Ballard et al., 1993) were manually dissected and truncated anterior to the first pair of pharyngeal pouches. The cephalic explant was transferred to O.C.T. medium (Tissue-Tek O.C.T. compound, Sakura Finetek), frozen in liquid nitrogen prior to sectioning (18 mm cryostat sections). Each frozen section was transferred into Trizol (200 μL) immediately after collection. Total RNA was extracted using standard Trizol extraction protocol and purified on Macherey Nagel NucleoSpin RNA XS columns according to the manufacturer’s instructions. 2 ml (1:200,000) spike-in RNA (ERCC) were added to each section prior RNA extraction as internal control.

### Illumina Library Construction and Sequencing

cDNA synthesis, amplification, and Illumina library construction were conducted following (Holler and Junker, 2019) with the following modifications. Superscript II reverse-transcriptase was replaced by SuperScript IV (Invitrogen) and cDNA clean-up was done using Omega Bio-Tek’s Mag-Bind TotalPure NGS. Sequencing was conducted on the DNB-seq platform (100 bp paired-end).

### Read Mapping and Counting

Reads were mapped onto the reference database of predicted genes (provided in Supplementary Table 1; annotation in Supplementary Table 2) complemented with spike-in sequences. Briefly, the construction of this database (to be reported elsewhere) involved building an isoform collapsed version of the NCBI gene predictions annotated from the catshark genome assembly^1^ and a subsequent extension of 3′ UTRs. The resulting gene models are referred to as genes hereafter for simplicity. Reference indexing, read mappings and quantifications were done using Kallisto v0.44.0 using the –bias parameter and bootstrapping 100 times. Read counts were aggregated for each gene and each sectioning plane, and normalized against the total spike-in read counts for each section. Ratios between spike-in input and output were calculated for the sum of all sections along each section plane.

### 3D Model Construction

To generate a 3D binary mask, a stage 17 head explant dissected as described above was fixed in PFA 4%, DAPI stained, dehydrated in methanol and cleared in Benzyl Alcohol/Benzyl Benzoate (1/2) prior to mounting and imaging with a SP8X confocal microscope (Leica). A 3D binary mask was built using Fiji to obtain a binary image of the tissue. It was then oriented transversally using Interactive Stack Rotation plugin and resized to match sectioning planes and section numbers along all three planes. The 3D expression genome-wide profile was reconstructed from normalized read counts for each gene and each section plane using the MATLAB code reported in Junker et al. (2014). In short, following the virtual partitioning of the binary mask in serial digital sections as described above, an iterative process was applied to 1D profiles of each gene in each sectioning plane, multiplying the 3D expression of the gene by said profile in each plane in succession. 3D profiles of selected genes were then visualized using Fiji. A custom Fiji macro was also written in order to get all 3D profiles, each viewed in all three sectioning planes, in the AVI format and with filenames indicating gene identification and annotation (.avi files for all genes available upon request).

### Statistical Analysis

For the autocorrelation analysis, Moran’s indexes were calculated for each gene on a volume defined as the voxels on adjacent sections on all three section planes, giving a 3 × 3 × 3 cube centered on each voxel examined. Supports for the indexes thus obtained were estimated by statistical tests, consisting in calculating the Pearson correlation between the expression of a gene between all possible pairs of neighboring voxels, using the R libraries spdep (especially moran.test) and ade4. Pearson correlations between the 3D expressions of each possible pair of genes in each voxel (18 μm cube at the intersection of one section of each sectioning plane) were calculated using the R command cor with default parameters.

### *In situ* Hybridization of Whole-Mount Embryos and Sections

Whole-mount ISH and ISH of paraffin sections were conducted using standard protocols as described previously (Lagadec et al., 2015).

## Results

### Obtaining Genome-Wide RNA Profiles for Serial Sections Along Antero-Posterior, Dorso-Ventral, and Left-Right Axes

**FIGURE 1:**
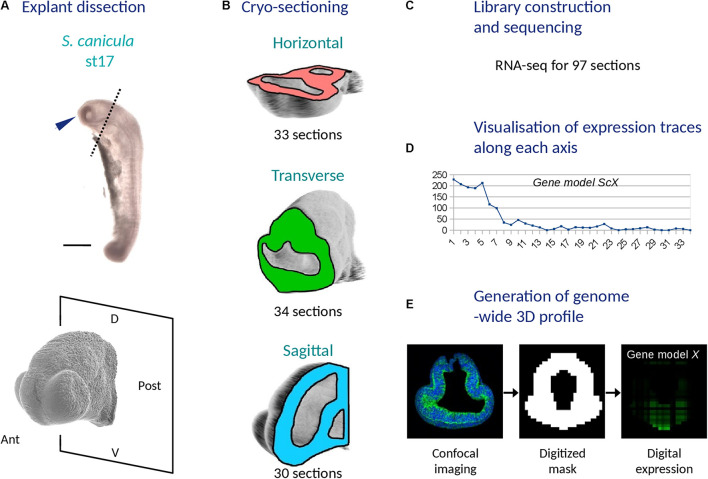
Main experimental steps used to generate a 3D RNA profile of the catshark embryonic head (stage 17). **(A)** Explant dissection: the top panel shows a left side photograph of a stage 17 catshark embryo with a blue arrowhead showing the dissected explant and a dotted line delimiting its posterior boundary and the bottom panel shows a higher magnification confocal view of the explant with the posterior dissection plane. **(B)** Cryo-sectioning: schemes show the planes used to generate series of cryo-sections (horizontal, pink; transverse, green; sagittal, blue). The total number of sections along each plane is indicated. **(C)** Library construction and sequencing resulting in RNA-seq data for a total of 97 sections. **(D)** Visualization of expression traces along each axis for all gene models. **(E)** Generation of a genome-wide 3D profile with its three steps (acquisition of a confocal 3D image of the explant, generation of a digitized model and computation of digital expressions for each voxel and gene model). D, dorsal; V, ventral; Ant, anterior; Post, posterior.

In order to obtain a digital transcriptomic map of the catshark stage 17 forebrain, we excised three embryonic head explants truncated anterior to the first branchial pouch (Figure 1A). Using cryostat sectioning along AP (antero-posterior), DV (dorso-ventral) and LR (left-right) axes, we obtained a total of 34 transverse, 33 horizontal, and 30 sagittal 18 mm frozen sections (Figure 1B). We next synthesized cDNA for each individual section using barcoded primers, which allowed to pool cDNA samples for linear amplification and quality controls of amplified products (Figure 1C and Supplementary Table 3). We next proceeded to Illumina library construction and sequencing to obtain about 210 million reads (PE-100). For the bioinformatic analysis, reads were demultiplexed and mapped onto a gene reference obtained taking advantage of the catshark genome NCBI annotation and of transcriptomic resources available in our laboratory (Supplementary Tables 1, 2), which led to the assignment of 76% reads to a reference contig (Supplementary Figures 1A–C,D–F,G–I). For each section plane, a minimum of 4 reads in at least two sections were detected prior to normalization for more than 13000 contigs (13354, 13501, and 14239 for sagittal, horizontal, and transverse planes, respectively). Read counts were then normalized, allowing the generation of expression profiles for any predicted gene along each section plane and of a genome-wide 3D digital model of gene expression (Figures 1D,E).

### Expression Traces Along Antero-Posterior, Dorso-Ventral, and Left-Right Axes Reflect Expression Restrictions of Candidate Regional Markers

**FIGURE 2:**
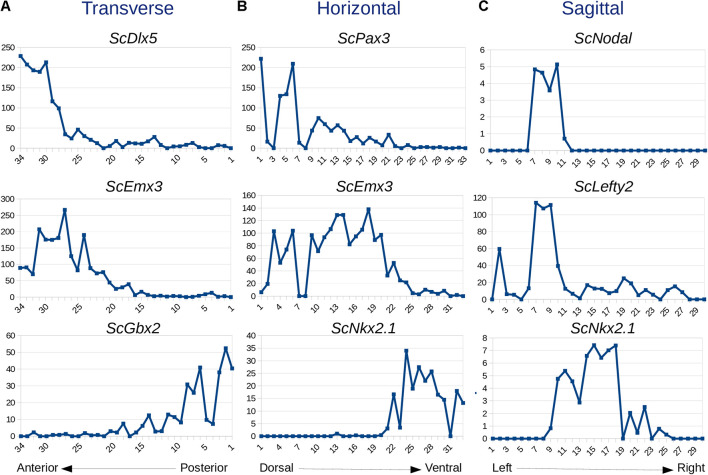
Expression traces for selected genes along transverse **(A)**, horizontal **(B)**, and sagittal **(C)** planes. For each graph, section numbers and corresponding read counts are indicated along x- and y-axes, respectively. Sections 1–34, 1–33, and 1–30 are, respectively, numbered from posterior to anterior in **(A)**, dorsal to ventral in **(B)**, and left to right in **(C)**. Gene names are indicated at the top of each profile.

In order to test whether variations in read counts between serial sections along each axis reflect gene regional expressions, we focused on forebrain markers previously characterized in the catshark (*Dlx5*, *Emx3*, *Gbx2*, *Pax3*, *Nkx2.1*, *Nodal*, *Lefty2*; Supplementary Table 4). Markedly higher *Dlx5* and *Emx3* read counts are found in anterior, versus posterior, transverse sections in line with their telencephalic expression. Read counts drop to basal levels from sections 1–27 for *Dlx5* and from sections 1–20 for *Emx3*, which accurately reflects the more restricted anterior territory of the former (Derobert et al., 2002; Santos-Durán et al., 2015; Figure 2A). In contrast, read counts peak at posterior levels (sections 1–8) for *Gbx2*, a midbrain marker (Figure 2A). Along the DV axis, *Pax3* and *Emx3* read counts are high dorsally and return to basal levels in more ventral sections, with a more dorsal peak for the former, while *Nkx2.1* reads are only detected in ventral-most sections (22–33), in line with ISH profiles for these genes (Derobert et al., 2002; O’Neill et al., 2007; Santos-Durán et al., 2015; Figure 2B). Along the left-right axis, sharp left restricted peaks are observed for *Nodal* and *Lefty2* (sections 7–10), consistent with the asymmetric left diencephalic territory reported at this stage (Lagadec et al., 2015; Figure 2C). In contrast, the curve shows a plateau at the level of sections located on each side of the midline (sections 10–18) for the symmetrically expressed hypothalamic marker *Nkx2.1* (Santos-Durán et al., 2015; Figure 2C).

### Construction of a 3D Digital RNA Profile Reproducing *in situ* Hybridization Expression Patterns

**FIGURE 3:**
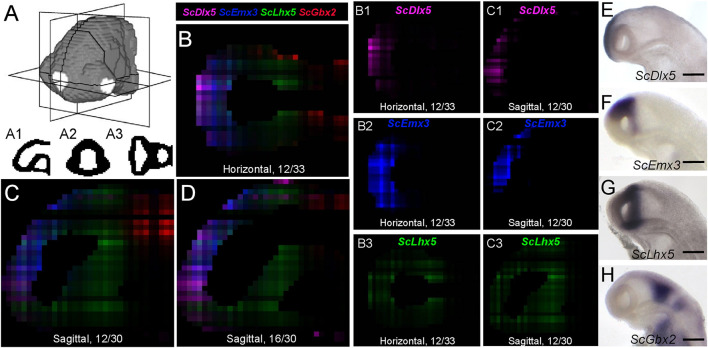
Digital profiles reproduce regional patterns along AP axis. **(A)** Scheme representing the digitized model of stage 17 catshark head with sagittal, transverse and horizontal digital section planes shown as white rectangles. **(A1**,**A2**,**A3)** show examples of digitized sections along these planes. **(B–D)** Digital horizontal **(B)** and sagittal **(C,D)** sections showing merged profiles of *ScDlx5* (magenta), *ScEmx3* (blue), *ScLhx5* (green), and *ScGbx2* (red). Sections are numbered from dorsal to ventral in panel **(B)** and from left to right in panels **(C,D)**. **(B1–3**,**C1–3)** Digital sections showing the individual profiles, respectively, merged in panels **(B**,**C)** for *ScDlx5* **(B1,C1)**, *ScEmx3* **(B2,C2)**, and *ScLhx5* **(B3,C3)**. **(E–H)** Left lateral views of stage 17 catshark embryonic heads after ISH with probes for *ScDlx5* **(E)**, *ScEmx3* **(F)**, *ScLhx5* **(G)**, and *ScGbx2* **(H)**. Scale bars = 200 μm.

**FIGURE 4:**
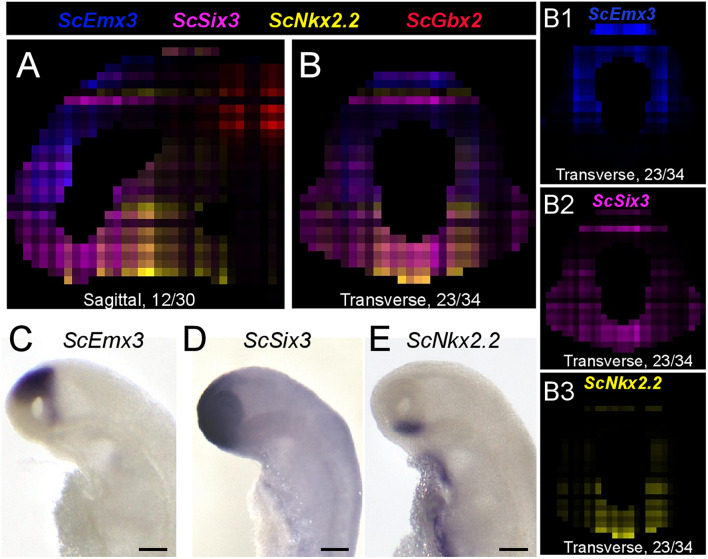
Digital profiles reproduce regional patterns along DV axis. **(A,B)** Digital sagittal **(A)** and transverse **(B)** sections showing merged profiles of *ScEmx3* (blue), *ScSix3* (magenta), *ScNkx2.2* (yellow), and *ScGbx2* (red). Sections are numbered from left to right in panel **(A)** and from posterior to anterior in panel **(B)**. **(B1–3)** Digital sections showing the individual profiles merged in panel **(B)** for *ScEmx3* **(B1)**, *ScSix3* **(B2)**, and *ScNkx2.2* **(B3)**. **(C–E)** Left lateral views of stage 17 catshark embryonic heads after ISH with probes for *ScEmx3* **(C)**, *ScSix3* **(D)**, and *ScNkx2.2* **(E)**. Scale bars = 200 μm.

**FIGURE 5:**
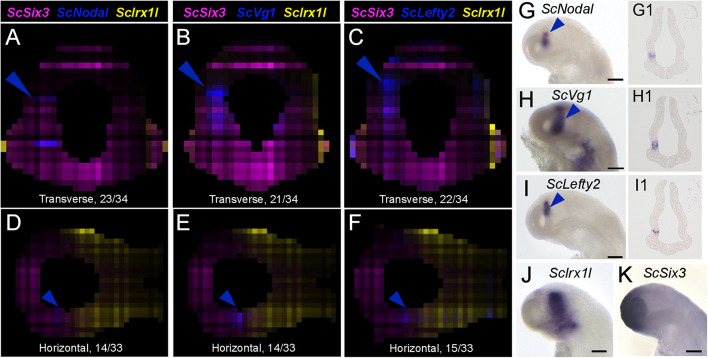
Nodal signaling components exhibit left enriched profiles. **(A–F)** Digital transverse **(A–C)** and horizontal **(D–F)** sections showing merged profiles of *ScSix3* (magenta), *ScIrx1l* (yellow), and in blue *ScNodal* **(A,D)**, *ScVg1* **(B,E)**, *ScLefty2* **(C,F)**. Sections are numbered from posterior to anterior in panels **(A–C)** and from dorsal to ventral in panels **(D–F)**. **(G–K)** Left lateral views of stage 17 catshark embryonic heads after whole-mount ISH with probes for *ScNodal* **(G)**, *ScVg1* **(H)**, *ScLefty2* **(I)**, *ScIrx1l* **(J)**, and *ScSix3* **(K)**. **(G1–I1)** Horizontal sections of the brain of stage 17 catshark embryos after hybridization with probes for *ScNodal* **(G1)**, *ScVg1* **(H1)**, *ScLefty2* **(I1)**. Blue arrowheads point to *ScNodal*, *ScVg1*, and *ScLefty2* left-restricted digital or ISH signals in the dorsal diencephalon. Scale bars = 200 μm.

We next projected the sequence data obtained for each section onto a mask imaged by confocal microscopy (Figure 1E) to generate a 3D RNA profile. To do so, we used iterative proportional fitting (Fienberg, 1970), as described in Junker et al. (2014). This resulted in a digital 3D model, containing expression information computed by the algorithm, referred to hereafter as digital expression, for every gene of the reference database and in every voxel identified by its (x, y, z) coordinates in the mask. In order to test this model and assess whether virtual expression patterns reflect expected ISH expression profiles, we focused on forebrain regional markers known to exhibit conserved and highly specific expression patterns across vertebrates, either along AP (antero-posterior), DV (dorso-ventral), or LR (left-right) axis (Figures 3–5; Supplementary Figure 2; Supplementary Table 4). Along the AP axis, we visualized digital profiles for *ScDlx5*, *ScEmx3*, *ScLhx5*, and *ScGbx2* (Supplementary Videos 1, 2). *ScDlx5* and *ScEmx3* exhibit anterior digital expressions, while *ScLhx5* positive voxels are located in a more medial position, adjacent to a posterior *ScGbx2* territory, in line with ISH expressions (compare Figures 3C,D,C1–C3 with Figures 3E–H). Similarly, as observed in ISH, *ScEmx3* digital territory extends further laterally and posteriorly than the one of *ScDlx5* (Figures 3B,B1,B2) and *ScLhx5* digital expression exhibits a sharp posterior border, contrasting with its anterior extension (Figures 3B3,C3). Similarly, along the DV axis, *ScNkx2.2* positive voxels are restricted to a medial and ventral location relative to optic evaginations, within a broader *ScSix3* digital territory (Figures 4A,B,B2,B3 and Supplementary Videos 3, 4). The latter overlaps with *ScEmx3* digital expression, which extends further posteriorly and is restricted dorsally (Figures 4A,B,B1,B2). These features reproduce those observed in whole-mount ISH (Figures 4C–E). Along the left-right axis, left-restricted (*ScNodal*, *ScVg1*) or left-enriched (*ScLefty2*) digital signals are observed dorsally and posteriorly to optic evaginations, at the interface between *ScSix3* anterior and *ScIrx1l* posterior digital territories, as in whole-mount ISH (compare Figure 5A–F to Figures 5G–K; Supplementary Videos 5–10). Digital signals are primarily located dorsally as expected for *ScNodal*, *ScVg1*, and *ScLefty2* (Figures 5A–C,G1–I1), but a few additional positive voxels, without clear counterparts in ISH, can be observed more ventrally at the level of optic evaginations (Figures 5A,C). The accuracy of the digital profiles was further assessed by analysis of digital expressions of additional markers including paralogous genes (Supplementary Figure 2 and Supplementary Videos 11–16). Comparison of *ScNkx2.1* and *Nkx2.2* digital profiles shows a broader expansion of the latter compared to its paralog along the AP axis, as observed in ISH (Supplementary Figures 2A,A1,A2). Along the same line, the sharp dorsal and ventral boundaries of *ScSix6* territory and its relative location within the broader *ScSix3* domain are reproduced by the digital profiles of the paralogs (compare Supplementary Figures 2B,B1 to Figures 2C,C1), as well as the respective expression characteristics of *ScFgf17* (restricted to the anterior-most part of the forebrain) and *ScFgf8* (harboring a major midbrain expression and a more diffuse anterior signal) (Supplementary Figure 2D; compare Supplementary Figures 2D1,2D2). The ventral expansion of forebrain markers such as *ScFoxg1* or *ScFezF2* relative to *ScEmx3* is also correctly predicted by digital profiles (compare Figure 3D with Supplementary Figures 2E,F,E1,E2,F1,F2).

### Autocorrelation as Estimate of the Spatial Restriction of Digital Profiles

**FIGURE 6:**
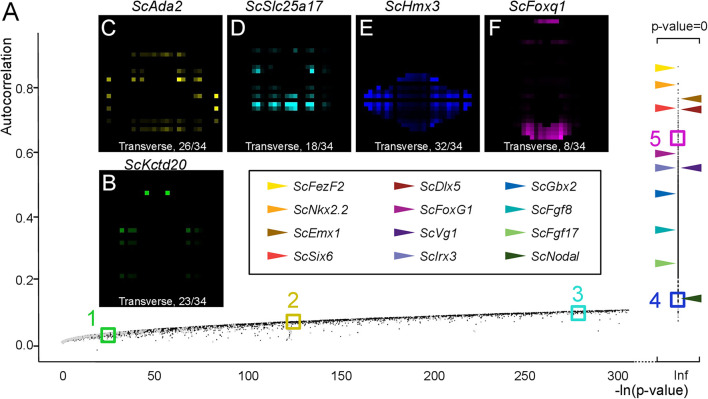
Autocorrelation as an indicator of profile regionalization. **(A)** Graph showing autocorrelation values (y-axis) and their statistical support [-ln(*p*-value) on x-axis] for all gene models. Each dot corresponds to a single gene model, in gray if the corresponding total digital expression is below 100, otherwise in black. Gene models with null *p*-values are shown on the right of the graph with –ln(*p*-value) = infinite (Inf). Horizontal arrowheads point to dots corresponding to a selection of genes, exhibiting clearly regionalized expressions in ISH experiments. Color codes for these genes are shown boxed in black. Colored boxes delimit sectors of the graph numbered from 1 to 5 and containing gene models with decreasing *p*-values/increasing autocorrelations (green, sector 1; yellow, sector 2, cyan, sector 3; blue, sector 4; magenta, sector 5). **(B–F)** Digital transverse sections showing examples of profiles observed in sectors 1 **(B)**, 2 **(C)**, 3 **(D)**, 4 **(E)**, and 5 **(F)**. The corresponding gene identities are indicated and sections are numbered from posterior to anterior.

In order to extract lists of genes exhibiting regionalized digital expression patterns, we computed spatial autocorrelations, commonly used to analyze the spatial structure of geographically distributed biological variables (Diniz-Filho et al., 2009) to the analysis of our digital expression model (Figure 6 and Supplementary Figure 3). The underlying rationale is that genes displaying regionalized profiles should tend to exhibit similar digital expressions in neighboring voxels within their expression territory and therefore show high autocorrelation values (Supplementary Figure 3A). Moran’s index, which provides a measure of spatial autocorrelation, and the *p*-value evaluating its statistical support, were calculated for all genes of the reference database (Figure 6A and Supplementary Table 5). In order to assess the relevance of this notion to the identification of regionalized genes, we first surveyed Moran’s indexes (i) and *p*-values obtained for a selection of the forebrain regional markers analyzed above, exhibiting discrete digital territories of variable size (*ScFezF2*, *ScNkx2.2*, *ScEmx3*, *ScSix6*, *ScDlx5*, *ScFoxg1*, *ScVg1*, *ScIrx3*, *ScGbx2*, *ScFgf8*, *ScFgf17*, *ScNodal*: Figure 6A). In all cases, indexes > 0.1, supported by null *p*-values, were retrieved. The lowest index is obtained for *ScNodal* (*i* = 0.11), which has a relatively low total digital expression (*n* = 619 counts over all voxels), while highest indexes are obtained for *ScFezF2*, *ScScNkx2.2*, and *ScEmx3*, three genes exhibiting high digital expressions (total digital expression summed over all voxels > 14000) and a single, continuous territory. Reciprocally, we randomly selected genes within 5 sectors of the *p*-value versus Moran’s index graph (sector 1: 0.01 < *i* < 0.02; sector 2: 0.06 < –*i* < 0.07; sector 3: 0.09 < *i* < 0.1; sector 4: *p*-value = 0; and 0.1 < *i* < 0.2: sector 5: *p*-value = 0 and 0.6 < *i* < 0.7; Figure 6A). All genes within sector 4 and 5 exhibited strongly regionalized digital territories, with a digital expression observed in all voxels in the former and a majority of voxels in the latter (Figures 6E,F and Supplementary Figures 4D,E). In contrast, labeled voxels appeared more and more dispersed and generally less numerous from sector 3 to 1 (Figures 6B–D and Supplementary Figures 4A–C). In general, genes of low total digital expression were over-represented among those with low autocorrelations and high *p*-values (Supplementary Table 6). For instance, more than 19% of the genes displaying –log(*p*-value) < 50 have a total digital expression < 100 and this value drops to, respectively, 2.1 and 0.1% for genes with *i* < 0.1 and *i* > 0.1, *p*-value = 0. Of note, 12100 genes, i.e., more than 60% of annotated genes, exhibit significant autocorrelation values (*i* > 0.1, *p*-value = 0; Supplementary Table 5 and Supplementary Figure 3B).

### A Correlation Statistical Approach Allows *de novo* Detection of Co-expressed Genes

**FIGURE 7:**
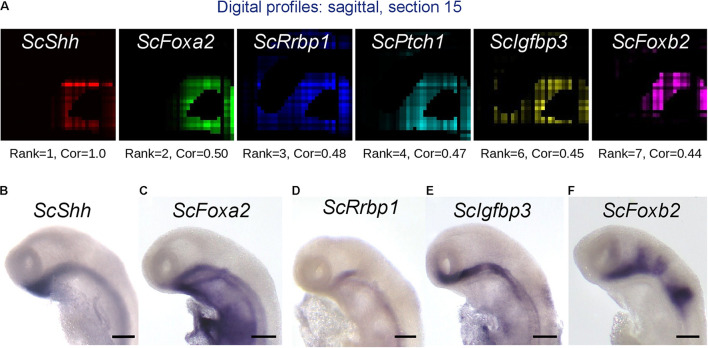
Identification of genes harboring expression correlation with *ScShh*. **(A)** Digital profiles on sagittal section number 15 (out of 30, from left to right) of a selection of genes showing a high expression correlation to *ScShh* (red), *ScFoxA2* (green), *ScRrbp1* (blue), *ScPtch1* (cyan), *ScIgfbp3* (yellow), and *ScFoxb2* (purple). The rank of each gene in the list of gene models ordered by decreasing correlation to *ScShh* and the correlation value (Cor) are indicated below each section. **(B–F)** Left lateral views of stage 17 catshark embryonic heads after ISH using probes for *ScShh* **(B)**, *ScFoxA2* **(C)**, *ScRrbp1* **(D)**, *ScIgfbp3* **(E)**, and *ScFoxb2* **(F)**. Scale bars = 200 μm.

The availability of a spatially resolved genome-wide profile allows the search for genes displaying specific expression criteria, for instance coexpression with selected subterritory markers. To exploit this possibility, we used a statistical approach relying on calculations of Pearson correlations (Cor) between pairs of genes (Supplementary Figure 5A). We first calculated Pearson correlations between digital expressions of all coding gene pairs taking over all voxels of our 3D model. Lists of genes ranked by correlation coefficient can then be extracted from this dataset for every single gene of the reference database. In order to assess the possibility to identify genes coexpressed with a given candidate by this method, we focused on correlations with *ScShh*, which shows a highly specific expression in the ventral midline, faithfully reproduced by the digital pattern, as proof-of-concept (Figures 7A,B and Supplementary Table 7). All 12 genes most strongly correlated to *ScShh* (Cor > 0.4) harbor a ventral digital expression, largely overlapping with the one of *ScShh* (Figure 7A and Supplementary Figure 5B). This list contains the catshark orthologs of two midline markers, respectively, coding for the Shh receptor Ptch1 and the extracellular matrix protein Slit3 (Ingham and McMahon, 2001; Yeo et al., 2001). In order to further validate the possibility of identifying novel genes coexpressed with *ScShh* by this approach, we conducted ISH for candidates retrieved from this list (*ScFoxa2*, *ScRrbp1*, *ScRop1l*, *ScIgfbp3*, *ScFoxb2*, *ScCcd39*). While no or very faint midline signals were observed for *ScRop1l* and *ScCcd39* (not shown), strong ventral restricted signals similar to *ScShh* were retrieved for *ScFoxa2*, as previously described (Ang and Rossant, 1994; Weinstein et al., 1994), but also for a set of genes not previously reported to display midline expression, such as *ScRrbp1*, *ScIgfbp3*, and *ScFoxb2* (Figures 7B–F). This validates both the predictive power of the 3D model and the possibility to extract novel information using a correlation approach.

## Discussion

Since the initial description of RNA tomography (Junker et al., 2014), this technique has been applied to a limited number of systems, including limbs, the regenerating or pathological heart and aorta formation, in major model organisms including the zebrafish, mouse, chick, and *Drosophila* (Wu et al., 2016; Combs and Eisen, 2017; Burkhard and Bakkers, 2018; van den Brink et al., 2020; Yvernogeau et al., 2020; Holler et al., 2021). Most of these analyses have focused on characterizations along a single plane of interest, which is sufficient to identify genes differentially expressed along a polarity axis but does not provide a 3D spatial resolution. Here, we have applied this technique for the first time to a chondrichthyan, the catshark *S. canicula*, focusing on the developing head, shortly after neural tube closure. The resulting dataset is a digital model of this structure, containing expression information in each voxel of this volume for about 30,000 sequences, of which 21,000 correspond to coding ones. It thus provides a resource of interest to address forebrain regionalization, sensory organ, placode, or diencephalic asymmetry formation in the catshark and explore the conservation of these processes across jawed vertebrates.

In this analysis, we find that 3D digital expressions for known markers of broad forebrain subdivisions faithfully reproduce the broad characteristics observed by ISH, with the method being sensitive enough to allow the detection of regionalized expression for transcription factors or signaling molecules. Although they do not reach cellular resolution, digital profiles also correctly predict expression details, including differences between paralogs, such as *ScNkx2.1* and *ScNkx2.2*, or *ScSix3* and *ScSix6*, or the asymmetric left/dorsal restriction of *Lefty2*/*Nodal*/*Vg1* in the diencephalon. In principle, a lower limit in resolution in this approach is imposed by section thickness, here 18 mm. Albeit in the same order of magnitude, this exceeds cell diameters in catshark stage 17 embryos, which range from 5 to 15 mm. Decreasing section thickness to 10 mm might be a first approach to improve resolution. Since the Cell-Seq2 protocol used for library construction is widely employed for single cell sequencing, this should not result in a marked loss in sensitivity due to the lower RNA quantity per section, pending a possible increase of the number of linear amplification cycles. Another factor limiting resolution and inherent to tomo-seq is related to the 3D *post hoc* reconstruction. The approach indeed integrates data from three independent, non-identical biological samples, sectioned along non-perfect orthogonal planes, and involves a projection on an independently generated digital model of the tissue analyzed. Manual curation of this digital model, aimed at adjusting its shape and size to the 1D profiles of previously characterized candidate markers along each axis, may alleviate this difficulty. However, tomo-seq is unlikely to reach cellular resolution and as such, cannot be applied to the analysis of non-replicable biological samples such as tumors biopsies, for instance. A panel of alternative techniques, reaching high spatial resolution in high throughput gene expression analyses of non-replicable specimens, have emerged in the past few years. Multiplexed fluorescent *in situ* hybridization (FISH) protocols, such as hybridization chain reaction (HCR) or single molecule FISH (smFISH) based methods, allow *in situ* expression analyses of tens to hundreds of candidate genes with cellular and even subcellular resolution, and they have been successfully applied to a variety of species and tissues (Chen et al., 2015; Choi et al., 2016, 2018; Moffitt et al., 2018; Trivedi et al., 2018; Andrews et al., 2020). Other approaches, relying on *in situ* RNA capture from histological sections and *ex situ* sequencing, provide unbiased and genome wide characterizations, albeit with slightly lower spatial resolution (Ståhl et al., 2016; Asp et al., 2019; Rodriques et al., 2019; Vickovic et al., 2019; Ortiz et al., 2020). Compared to these technologies, tomo-seq presents a number of distinctive advantageous features including (1) a robust and fast protocol, without the need for lengthy imaging processes or experimental optimizations aimed, for instance, at monitoring the balance between *in situ* RNA accessibility and diffusion in the tissue, (2) the use of standard, inexpensive reagents and equipment for library construction, and (3) the production of genome-wide expression data, which can be screened rapidly for thousands of candidate genes, possibly following filtering on expression level and autocorrelation as described here. The combination of this technique with other approaches reaching cellular resolution, such as scRNA-seq, or FISH based methods applied to panels of selected genes, may be powerful approaches for comprehensive characterizations of tissue or organ RNA profiles (van den Brink et al., 2020).

An important perspective opened by the availability of this resource is the possibility to identify new genes characterized by a defined expression feature, such as co-expression with a territory marker. Established methodologies are still lacking for 3D tomo-seq datasets, which have been thus far only produced in the zebrafish and mouse. Junker et al. (2014) previously reported that a euclidean distance-based method could lead to the detection of new genes displaying expression similar to an organizer marker. Here we used an alternative statistical method, relying on estimates of Pearson correlations between any pair of genes and over all voxels, to address this question. This approach retrieves genes known or expected to be co-expressed with *Shh* such as *Foxa2*, *Ptch1*, or *Slit3*, with high correlation values. It also identifies genes, such as *Rrbp1* or *Igfbp3*, that have to our knowledge not previously been reported as co-expressed with *Shh.* We also validate some of these profiles by ISH, which demonstrates the potential of this approach to explore the entire repertoire of genes expressed in territories of interest. Such exhaustive characterization is important to identify synexpression groups of genes possibly submitted to the same regulatory framework, but also to assess territory homologies based on a significant number of signature markers in comparative approaches.

Similarly, we used spatial autocorrelation as a measure to identify genes exhibiting regionalized expression. The underlying mathematical notion has been widely used in biology (Diniz-Filho et al., 2009), albeit not to tomo-seq data. Its adequacy in this context is related to the assumption that expression values per voxel are non-independent variables, neighboring voxels, for instance around a peak or trough, tending to behave similarly. This characteristic is indeed observed for most regionalized genes, albeit not for those exhibiting salt-and-pepper expression patterns, such as Notch targets (Caprioli et al., 2002). In line with the relevance of this indicator, strongly supported positive autocorrelation values (*p*-value ∼ 0) were obtained for all highly regionalized genes analyzed, whether expressed in broad territories like most of the forebrain markers tested, or in highly restricted domains such as left-restricted *Vg1* or *Nodal*. Reciprocally, randomly selected patterns exhibit an expression dispersal per voxel, which increases inversely proportionally to their autocorrelation value. These data point to autocorrelation as a relevant indicator to identify genes of regionalized expression. Its use suggests that as previously proposed (Junker et al., 2014), regionalized genes (*p*-value = 0, autocorrelation > 0.1) represent an important fraction of the total gene repertoire.

In summary, our results show that tomo-seq is an effective method to characterize broad partitions of organs or embryonic structures. The recurrent need for such analyses in the evo-devo field does not necessarily require cellular resolution. One limitation, however, is that the size of the tissue of interest be sufficient for a section-based approach: tomo-seq would, for instance, be inadequate for a similar forebrain characterization shortly after neural tube closure in the zebrafish, due to the small size of its embryonic head at this stage. More generally, large body size is an obvious limitation for the maintenance of large populations under laboratory conditions. This characteristic has thus been counter-selected in the choice of major model organisms in developmental biology, such as *Drosophila*, zebrafish, or *C. elegans*. However, large size becomes an advantage for section-based spatial transcriptomics techniques such as tomo-seq. These techniques provide novel tools in evo-devo models harboring large embryo and body sizes such as the catshark, which although non-amenable to genetic approaches, has become a reference for questions pertaining to the origin and ancestral characteristics of jawed vertebrates.

## Data Availability Statement

The datasets presented in this study can be found in online repositories. The names of the repository/repositories and accession number(s) can be found below: NCBI (accession: PRJNA758756).

## Ethics Statement

Ethical review and approval was not required for the animal study because the work only used non-mammalian embryos, at a stage preceding central nervous system differentiation. This study does not require approval from an animal ethics committee according to national and EU regulations.

## Author Contributions

RL, AQ, ML, AM, and LM conducted the experimental work (sectioning, Illumina library construction, imaging, and ISH). HM, KM, and SD took charge in bioinformatics (sequence processing and analysis, 3D reconstruction) and statistical analyses. SM, RL, and PB conceived the work, analyzed the data, and wrote the manuscript with inputs from all authors. All authors had full access to all data, approved the article, and took responsibility for the integrity of the data and the accuracy of the data analysis.

## Conflict of Interest

The authors declare that the research was conducted in the absence of any commercial or financial relationships that could be construed as a potential conflict of interest.

## Publisher’s Note

All claims expressed in this article are solely those of the authors and do not necessarily represent those of their affiliated organizations, or those of the publisher, the editors and the reviewers. Any product that may be evaluated in this article, or claim that may be made by its manufacturer, is not guaranteed or endorsed by the publisher.

## Footnotes

1
https://www.ncbi.nlm.nih.gov/assembly/GCF_902713615.1/#/st_Primary-Assembly
