# Supplementary material for: When Bigger Is Better: 3D RNA Profiling of the Developing Head in the Catshark Scyliorhinus canicula
Source: Front Cell Dev Biol. 2021 Oct 22;9:744982. doi: 10.3389/fcell.2021.744982 (PMC8569936; doi:10.3389/fcell.2021.744982)
Supplement: Supplementary file 1 [file Data_Sheet_3.PDF]

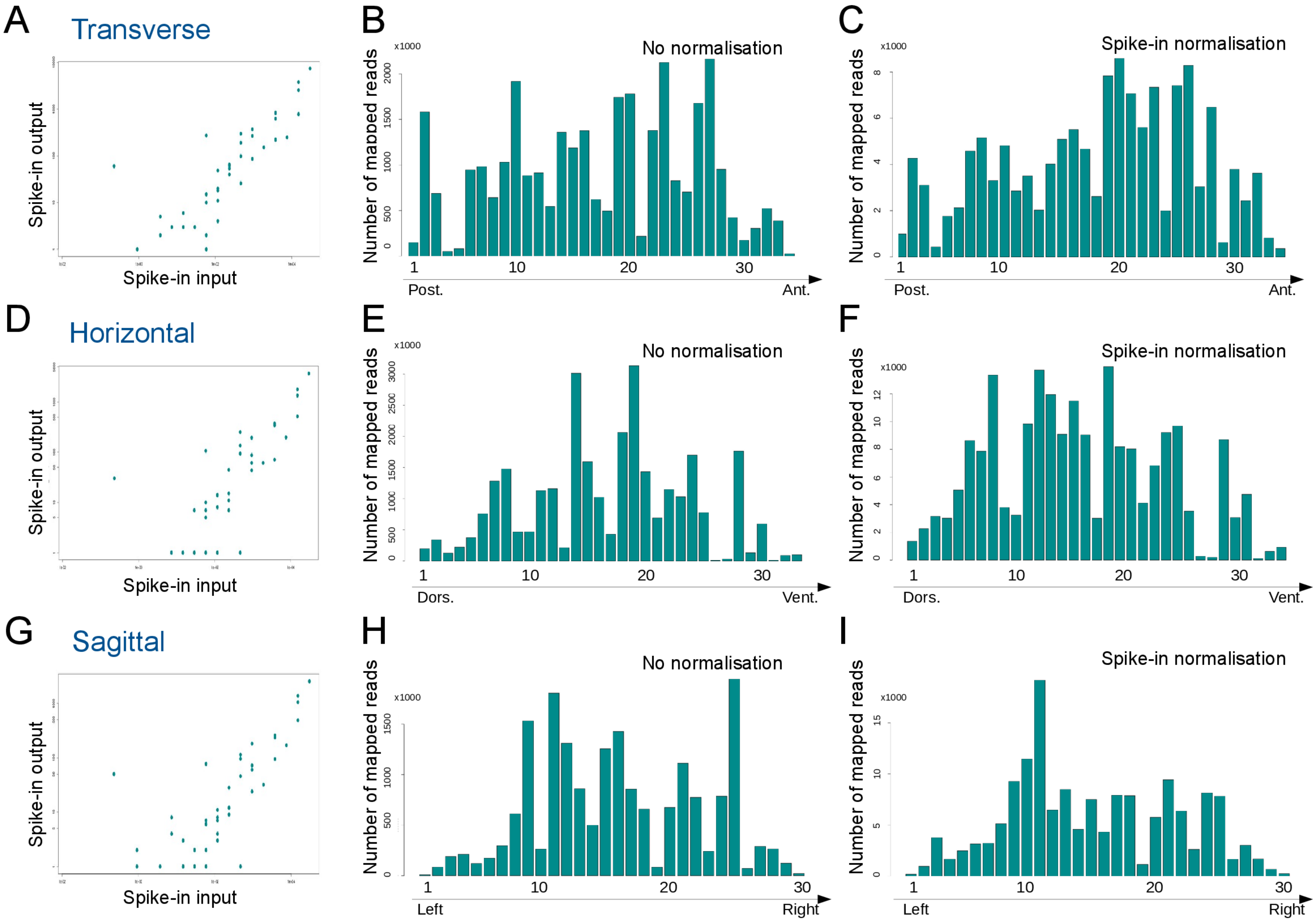

Supplementary Figure 1. General sequence analysis of sections. (A,D,G) Input/output relationship for spike-in RNA, summed over all transverse (A), horizontal (D) and sagittal (G) sections. Each dot corresponds to a spike-in form. Linearity is observed, suggesting a minimal amplification bias. **(B-C,E-F,H-I)** Number of reads mapping onto the gene model reference without normalisation (B,E,H) or following normalisation on spike-in (C,F,I). (B,C), (E,F) and (H,I) correspond to transverse, horizontal sections and sagittal sections respectively. Sections are shown and numbered from posterior (Post.) to anterior (Ant.) in (B-C), from dorsal (Dors.) to ventral (Vent.) in (E-F) and from left to right in (H-I).

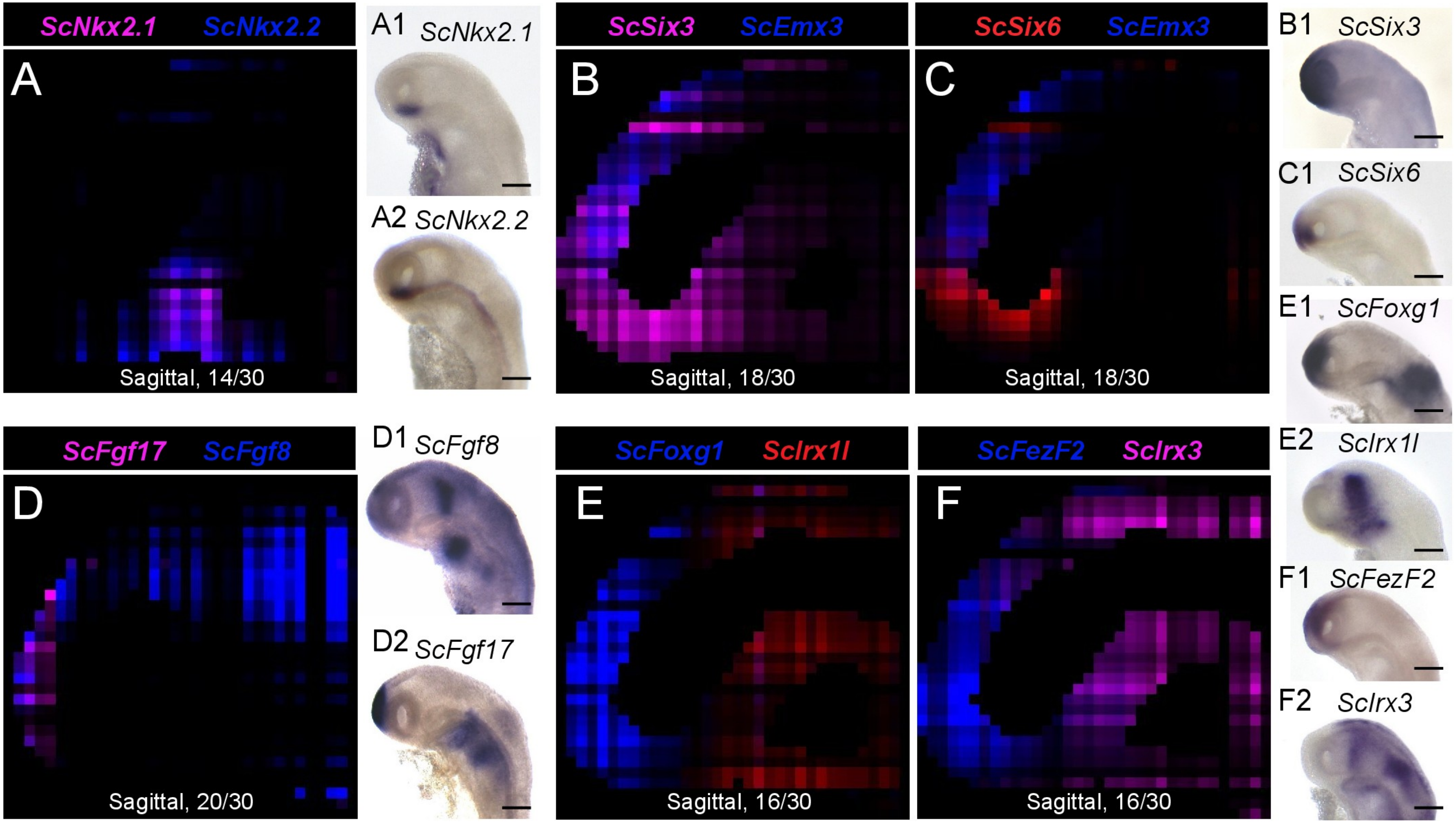

Supplementary Figure 2. Accuracy of 3D RNA profiles. (A-F) Digital sagittal sections showing merged profiles for: (A), *ScNkx2.1* (magenta) and *ScNkx2.2* (blue); (B), *ScSix3* (magenta) and *ScEmx3* (blue); (C), *ScSix6* (red) and *ScEmx3* (blue); (D), *ScFgf17* (magenta) and *ScFgf8* (blue); (E), *ScFoxg1* (blue) and *ScIrx1l* (red); (F), *ScFezF2* (blue) and *ScIrx3* (magenta). Sections are numbered from left to right. **(A1,A2,B1,C1,D1,D2,E1,E2,F1,F2)** Left lateral views of stage 17 catshark embryonic heads after ISH with probes for respectively *ScNkx2.1*, *ScNkx2.2*, *ScSix3*, *ScSix6*, *ScFgf8*, *ScFgf17*, *ScFoxg1*, *ScIrx1l*, *ScFezF*2 and *ScIrx3*. Scale bars=200µm.

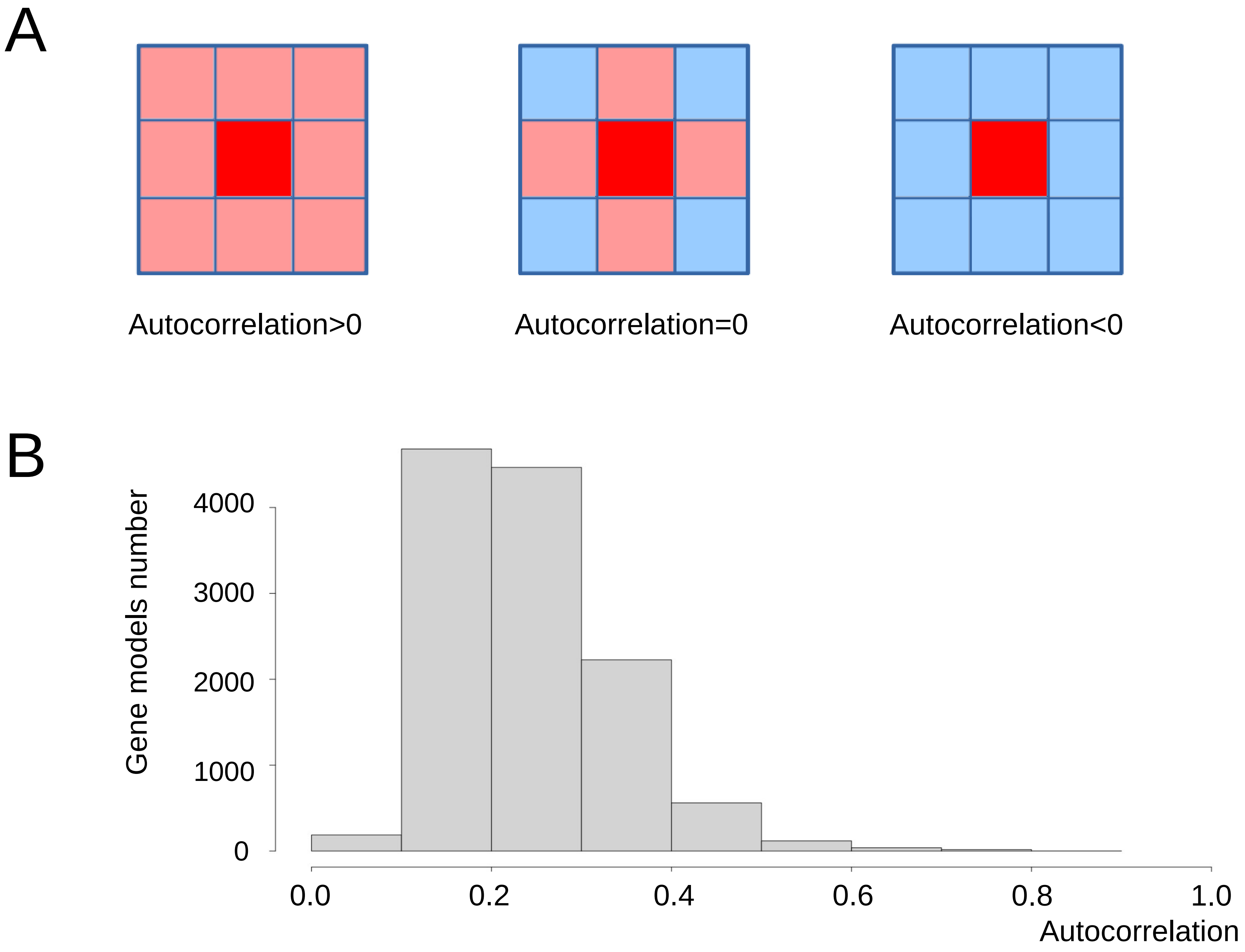

Supplementary Figure 3. Autocorrelation as a measure of expression regionalisation. (A) Schemes illustrating how local variations affect autocorrelation values. **(B)** Histogram showing the distribution of gene models depending on autocorrelation values for null p-values.

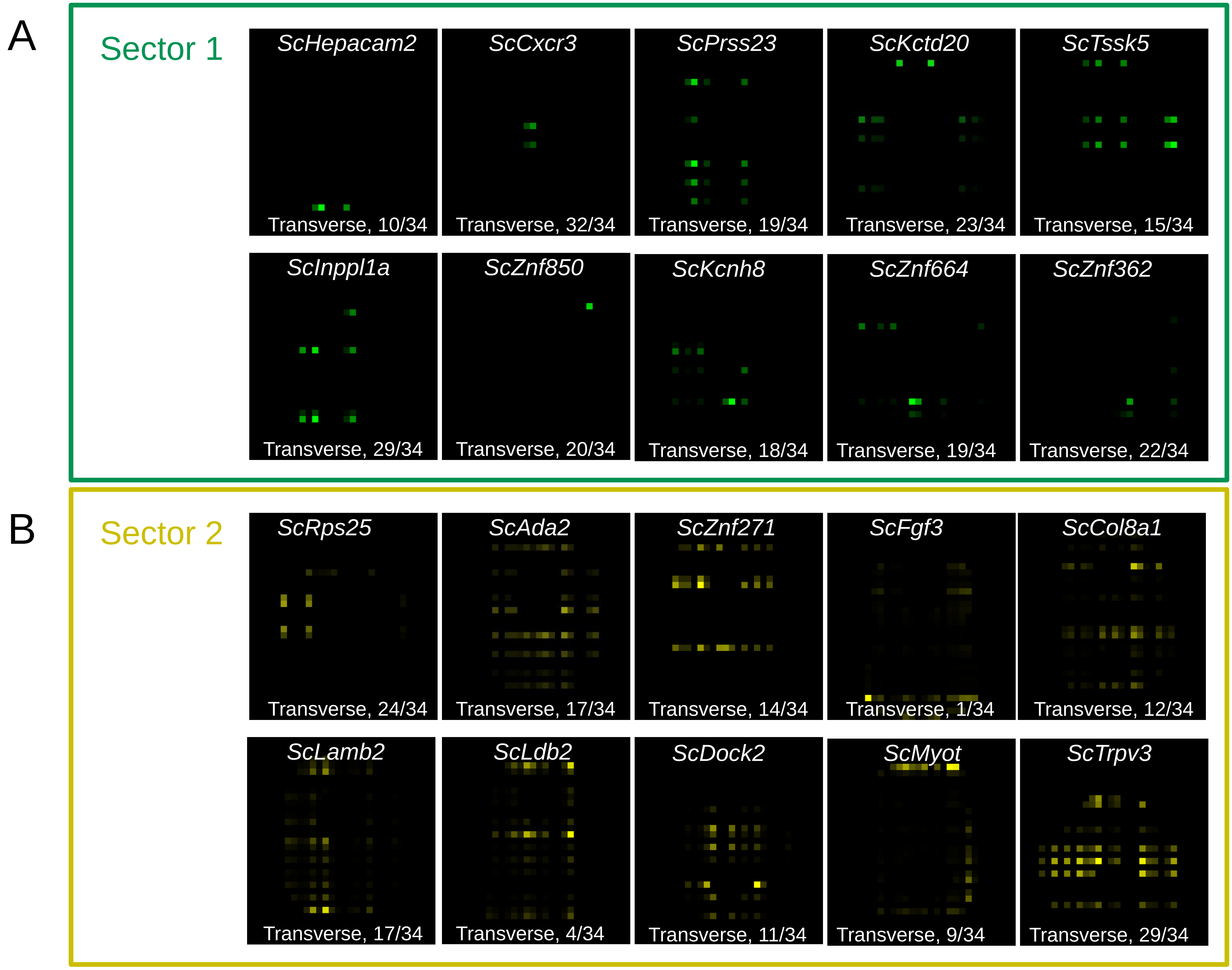
A
Sector 1
ScHepacam2
Transverse, 10/34
ScCxcr3
Transverse, 32/34
ScPrss23
Transverse, 19/34
ScKctd20
Transverse, 23/34
ScTssk5
Transverse, 15/34
ScInppl1a
Transverse, 29/34
ScZnf850
Transverse, 20/34
ScKcnh8
Transverse, 18/34
ScZnf664
Transverse, 19/34
ScZnf362
Transverse, 22/34
B
Sector 2
ScRps25
Transverse, 24/34
ScAda2
Transverse, 17/34
ScZnf271
Transverse, 14/34
ScFgf3
Transverse, 1/34
ScCol8a1
Transverse, 12/34
ScLamb2
Transverse, 17/34
ScLdb2
Transverse, 4/34
ScDock2
Transverse, 11/34
ScMyot
Transverse, 9/34
ScTrpv3
Transverse, 29/34

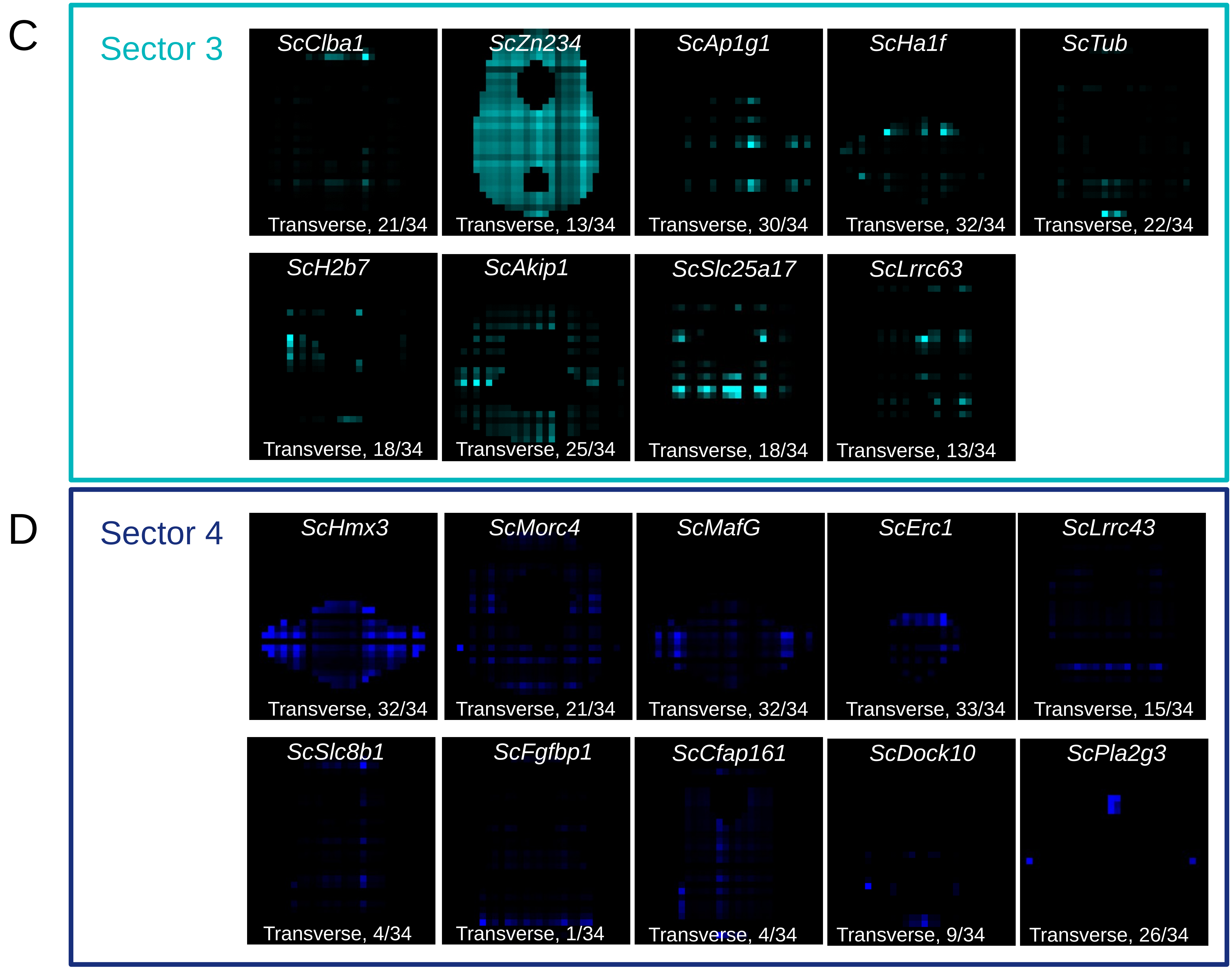

C
Sector 3
ScClba1
Transverse, 21/34
ScZn234
Transverse, 13/34
ScAp1g1
Transverse, 30/34
ScHa1f
Transverse, 32/34
ScTub
Transverse, 22/34
ScH2b7
Transverse, 18/34
ScAkip1
Transverse, 25/34
ScSlc25a17
Transverse, 18/34
ScLrrc63
Transverse, 13/34
D
Sector 4
ScHmx3
Transverse, 32/34
ScMorc4
Transverse, 21/34
ScMafG
Transverse, 32/34
ScErc1
Transverse, 33/34
ScLrrc43
Transverse, 15/34
ScSlc8b1
Transverse, 4/34
ScFgfbp1
Transverse, 1/34
ScCfap161
Transverse, 4/34
ScDock10
Transverse, 9/34
ScPla2g3
Transverse, 26/34

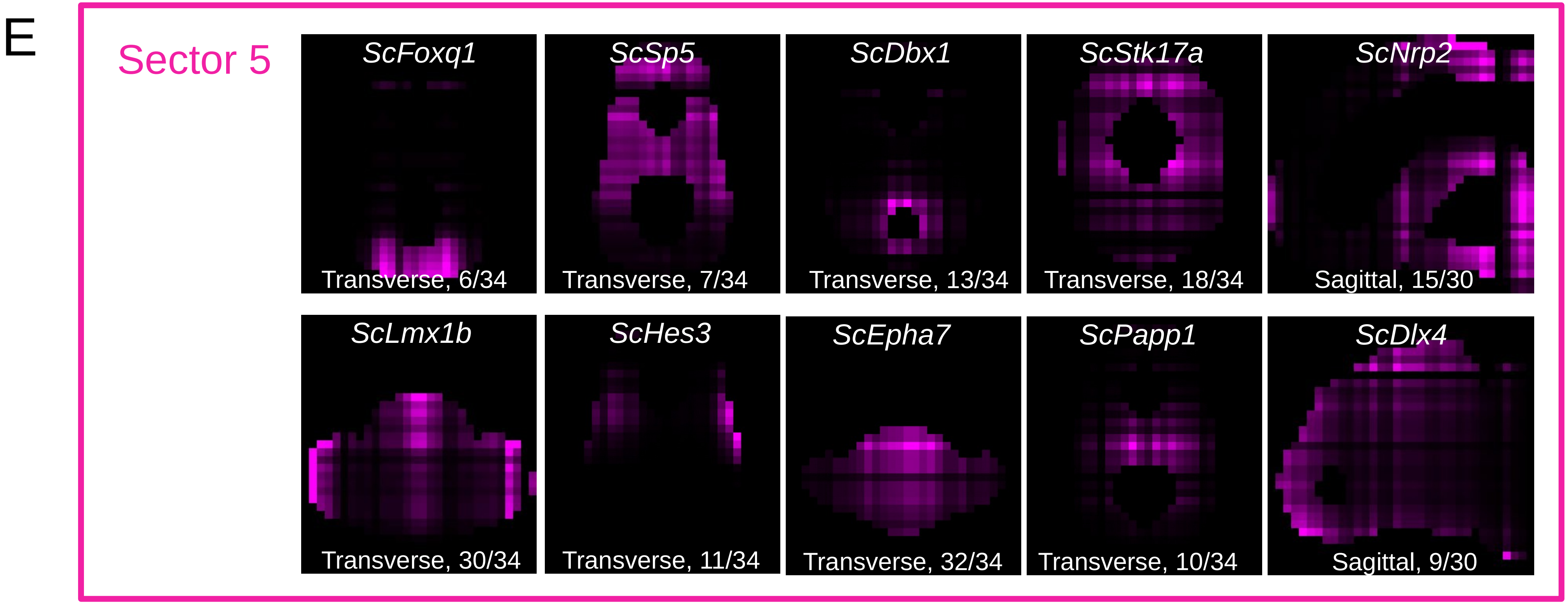

Supplementary Figure 4. Typical digital profiles obtained for gene models chosen randomly in selected sectors of the autocorrelation versus p-value graph. Digital sections showing the profiles of gene models found in sectors 1, 2, 3, 4 and 5 (as defined in Figure 6) are presented in (A), (B), (C), (D) and (E) respectively. Voxels harbouring digital expressions appear clustered in sectors 1 and 2, but more and more dispersed in sectors 3 to 1. Only one exception to this trend is observed (*ScZn234*, sector 3), expression being ubiquitous in this case.

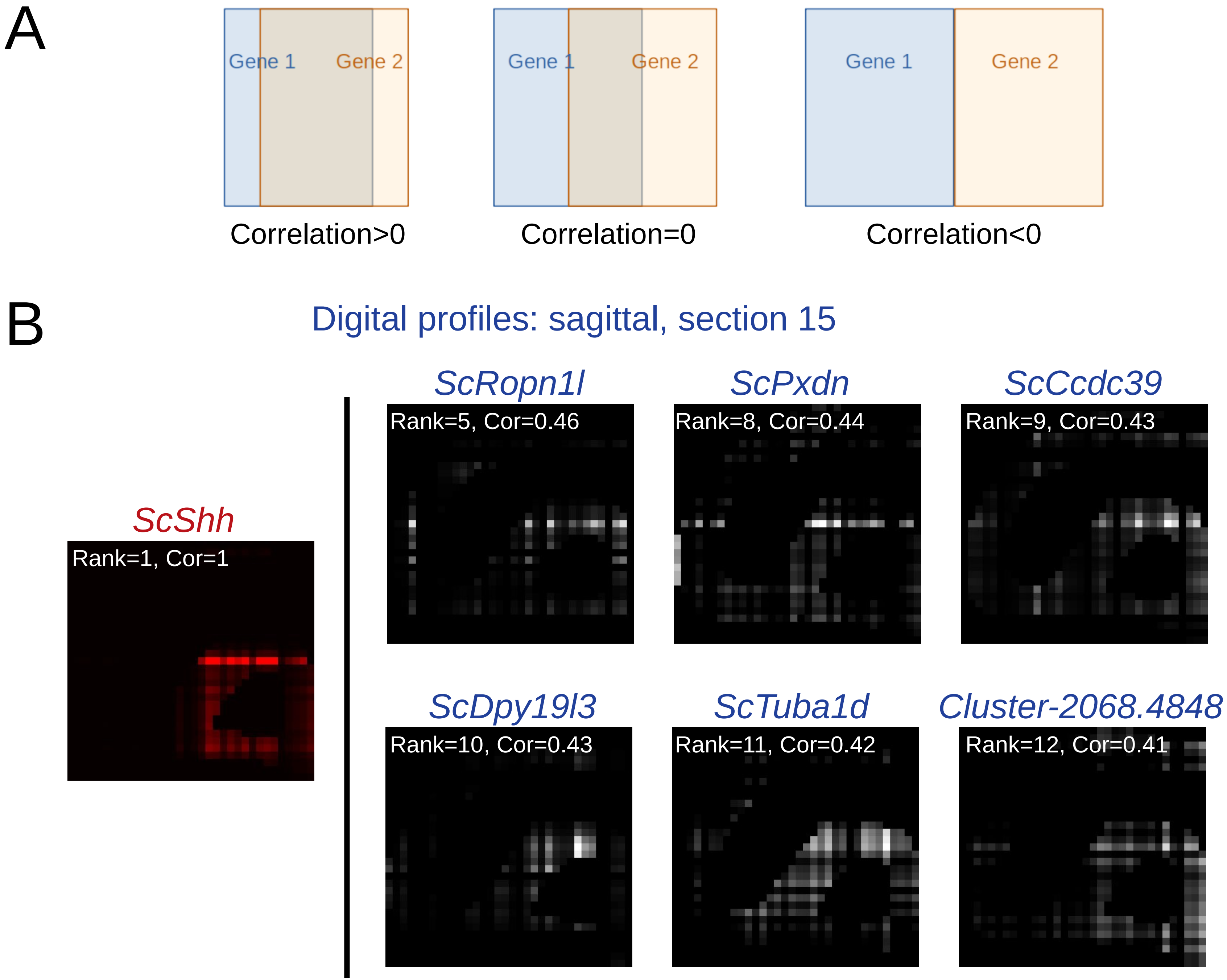

Supplementary Figure 5. Correlation as a measure of expression profile similarity. (A) Schemes illustrating positive, null and negative correlations between two genes. **(B)** Digital sagittal sections (section 15, from left to right) showing the profiles of genes showing high expression correlations to ScShh. The rank in the list of gene models ordered by decreasing correlation is shown, as well as the correlation value obtained for each gene shown. All gene models share a ventrally restricted signal similar to the one observed for *ScShh*.

Legends to Supplementary Tables and Supplementary Videos

Supplementary Table 1. Fasta file of the gene models database used for read mapping. The table contains the identity and sequence of the 30348 gene models used as reference in this study.

Supplementary Table 2. Annotation file of the gene models. The table contains 20554 gene models that could be annotated. For each gene model (column A), the identity of the best Blastx hit in Swissprrot is indicated in column B. Columns C and D respectively contain the query deduced amino acid and hit protein sequence contained within the alignment.

Supplementary Table 3. Primers used for library constructions and read counts obtained per section. For each section (column A), the names and sequences of the barcoded primers used for reverse transcription are indicated in columns B and C. The names and sequences of Illumina indexes used for Illumina library construction are listed in columns D and E. The read counts obtained after sequencing are indicated for each section in column F.

Supplementary Table 4. Identifiers of analyzed selected genes. Column 2 provides identifiers for the catshark genes, whose digital profiles were visualized and analyzed. For the genes analyzed by *in situ* hybridization, the sequences used as probes are shown in column 3.

Supplementary Table 5. Autocorrelation values for all gene models. Gene models are listed in column A, with their annotation in column B. The autocorrelation value and corresponding statistical support (p-value) are shown in columns C and D respectively. The total digital expression (summed over all voxels) is given in column E.

Supplementary Table 6. Effect of total digital expression level on autocorrelation. The table shows the number of gene models exhibiting a total digital expression <100 or >100 (columns B and C respectively; total in column C) depending on their autocorrelation/p-value (column A). Corresponding ratios are shown in columns E and F respectively.

Supplementary Table 7. Correlations with *ScShh*. For all gene models (column A, annotation in column B), 3D profile correlations with *ScShh* are shown in column C.

Supplementary Videos 1 and 2. Avi files containing merged digital profiles for *ScDlx5* (magenta), *ScEmx3* (blue), *ScLhx5* (green) and *ScGbx2* (red) along serial horizontal sections 1 to 33 (numbered from dorsal to ventral; Suppl. Video 1) and along serial sagittal sections 1 to 30 (numbered from left to right; Suppl. Video 2). Individual files can be opened in Fiji and visualized following appropriate size adjustment (interpolation: none).

Supplementary Videos 3 and 4. Avi files containing merged digital profiles for *ScSix3* (magenta), *ScEmx3* (blue), *ScNkx2.2* (yellow) and *ScGbx2* (red) along serial sagittal sections 1 to 30 (numbered from left to right; Suppl. Video 3) and along serial transverse sections 1 to 34 (numbered from posterior to anterior; Suppl. Video 4). Individual files can be opened in Fiji and visualized following appropriate size adjustment (interpolation: none).

Supplementary Videos 5-10. Avi files containing merged digital profiles for *ScSix3* (magenta), *ScIrx1l* (yellow), *ScNodal* (blue, Suppl. Videos 5 and 6), ScVg*1* (blue, Suppl. Videos 7 and 8) and *ScLefty2* (blue; Suppl. Videos 9 and 10). Suppl. Videos 5, 7 and 9 show serial transverse sections 1 to 34, numbered from posterior to anterior. Suppl. Videos 6, 8 and 10 show serial horizontal sections 1 to 33 (numbered from dorsal to ventral). Individual files can be opened in Fiji and visualized following appropriate size adjustment (interpolation: none).

Supplementary Videos 11-16. Avi files containing merged digital profiles along serial sagittal sections 1 to 30 (from left to right) for: Suppl. Video 11, *ScNkx2.1* (magenta) and *ScNkx2.2* (blue); Suppl. Video 12, *ScSix3* (magenta) and *ScEmx3* (blue); Suppl. Video 13, *ScSix6* (red) and *ScEmx3* (blue); Suppl. Video 14, *ScFgf17* (magenta) and *ScFgf8* (blue); Suppl. Video 15, *ScFoxg1* (blue) and *ScIrx1l* (red); Suppl. Video 16, *ScFezF2* (blue) and *ScIrx3* (magenta). Individual files can be opened in Fiji and visualized following appropriate size adjustment (interpolation: none).
